# Breaking the Cybernetic Code: Understanding and Treating the Human Metacognitive Control System to Enhance Mental Health

**DOI:** 10.3389/fpsyg.2019.02621

**Published:** 2019-12-12

**Authors:** Adrian Wells

**Affiliations:** ^1^School of Psychological Sciences, Faculty of Biology, Medicine and Health, The University of Manchester, Manchester, United Kingdom; ^2^Greater Manchester Mental Health NHS Foundation Trust, Manchester, United Kingdom

**Keywords:** metacognitive therapy, metacognition, self-awareness, transdiagnostic mechanisms, cognitive behavior therapy, neural networks, embodiment, attention

## Abstract

The self-regulatory executive function (S-REF) model explains the role of strategic processes and metacognition in psychological disorder and was a major influence on the development of metacognitive therapy. The model identifies a universal style of perseverative negative processing termed the cognitive attentional syndrome (CAS), comprised of worry, rumination, and threat monitoring in the development of disorder. The CAS is linked to dysfunctional metacognitions that include beliefs and plans for regulating cognition. In this paper, I extend the theoretical foundations necessary to support further research on mechanisms linking metacognition to cognitive regulation and effective treatment. I propose a metacognitive control system (MCS) of the S-REF that can be usefully distinguished from cognition and is comprised of multiple structures, information, and processes. The MCS monitors and controls activity of the cognitive system and regulates the behavior of neural networks whose activities bias the way cognition is experienced. Metacognitive information involved in the regulation of on-line processing includes metacognitive beliefs, metacognitive procedural commands, and more transient cybernetic code. Separation of the cognitive and metacognitive systems and modeling their relationship presents major implications concerning what should be done in therapy and how it should be done. The paper concludes with an in-depth consideration of methods that strengthen the psychological basis of psychotherapy and aid in understanding and applying metacognitive therapy in particular. Finally, limitations of the model and implications for future research on self-awareness, self-regulation, and metacognition are discussed.

## Introduction

Throughout the last 25 years, the Self-Regulatory Executive Function (S-REF) model (Wells and Matthews, 1994, 1996) has stimulated a large volume of research on cognitive control processes in psychological disorder and is the grounding of an effective psychological treatment: metacognitive therapy (MCT: Wells, 1995, 2009). In this paper, I consider the central principles of the model in light of recent evidence and expand on the functional components of its metacognitive control system. The aim is to provide a theoretical framework to stimulate and advance future research on varieties of metacognitive information, processes, and structures in psychological disorder, self-awareness, and treatment.

## Historical Context of the Self-Regulatory Executive Function Model

Our initial aim in the work leading to the S-REF was to take a robust scientific approach that was deeply rooted in cognitive psychology to develop an explanation of the mechanisms behind psychological disorder. That aim culminated in our book, *Attention and Emotion: A Clinical Perspective*; first published in 1994 and since re-published (Wells and Matthews, 1994, 2015). Our goal was to generate testable theory-based predictions that would lead to clinical innovation.

The S-REF model aimed to explain laboratory-based data on attention bias, individual differences in stress responses, and the cause of psychological disorder. This did not turn out to be a simple task, but it was a controversial one. The prevailing view at the time was that psychological disorder was largely an effect of bottom-up (automatic) stimulus-driven biases in processing resulting from schemas or associative networks. We questioned this view, setting out a model based on alternative mechanisms, involving maladaptation in top-down volitional cognitive control, arguing that clinical disorder is associated with a reduction in dynamic control and adaptability.

The application of cognitive psychology principles in the field of psychopathology and treatment was limited when we began. Innovative research on attention in anxiety (Mathews and MacLeod, 1985, 1986; Williams et al., 1988; Mathews et al., 1990; MacLeod, 1991) demonstrated that patients are characterized by a bias toward information with negative content. Our initial goal was to attempt to explain such selective processing. What might lead the emotional disordered patient to focus on negative information? We began by evaluating the success of existing theory in accounting for biased attention and its success in accommodating important attention factors; capacity limitation and distinctions between voluntary and involuntary (automatic) processes.

Influential models of psychological disorders centered on memory structures (e.g. schemas or associative networks) as key causes of disorder and the major treatment approaches focused primarily on the content of these structures and related cognitions. For example, Beck’s cognitive theory (Beck, 1976; Beck et al., 1985) of emotional disorders assigned a prominent role to the content of beliefs and interpretations in disorder, identifying the negative triad in depression and a preponderance of thoughts about danger in anxiety (e.g. “I’m going to physically collapse”). In contrast, we argued that maladaptation occurs principally due to volitional biases in executive control, in the selection of self-regulation strategies; the emotionally vulnerable person selecting those strategies that prolonged rather than terminated negative processing. Increasingly, we became aware of limitations of the schema and “automaticity” concepts as an explanation of these features of processing. In particular, they failed to account for the individuals influence over whether or not to continue with current processing. For instance, the content of self-knowledge or schemas (e.g. “I’m a failure as a mother”) does not explain bias in attention or cognitive regulation because the individual retains choice in whether or not to continue analyzing their failures. In effect, the role of top-down or executive processes in the regulation of processing necessitated elaboration. Therefore, our model aimed to explain how voluntary (executive processes) and involuntary processes interacted with stored knowledge, especially *metacognition* in the regulation of processing.

Metacognition refers to the structures, content, and processes involved in the monitoring, appraisal, and control of cognition. Sometimes loosely defined as that part of cognition that is turned onto itself, this simple definition may be misleading, because it suggests a single structure of cognition responsible for cognition and metacognition. Seminal work on metacognition prior to the S-REF model was predominantly in developmental, educational, and memory psychology with defining contributions of Flavell (1979), Nelson and Narens (1990), and colleagues.

In order to develop a comprehensive model of cognitive control and the prioritizing of negative processing, we predicted a central contribution of dysfunctional metacognition and attentional control plans stored in long term memory. Subsequently, the metacognitive component of the model was elaborated as the basis for metacognitive therapy (Wells, 1995, 2000, 2009), and the model was extended with greater detail of features of its architecture and metacognitive components (especially metacognitive beliefs). However, the central tenets of the theory and its implications, emphasizing universal top-down influences, remain the same.

The S-REF model has influenced the development of other treatment approaches. For example, Clark and Wells (1995) advanced a model and treatment of social phobia that has proven effective (Clark et al., 2006; Nordahl et al., 2016) and is a recommended intervention in health guidelines (NCCMH, 2013). Wider influences of the S-REF on psychotherapy are apparent as extensions of CBT, for example, “emotional schema” theory and treatment (Leahy, 2015). While in a separate line of work, metacognition has been formulated differently by Dimaggio et al. (2015) in their therapeutic approach of interpersonal therapy in personality disorder and by Moritz and Woodward (2007) in metacognitive training for schizophrenia.

## Outline of the Self-Regulatory Executive Function Model

The S-REF model is based on the principle that most psychological disorders are the result of a universal style of cognition and behavior termed the Cognitive Attentional Syndrome (CAS). The CAS is a state of processing where negative self-relevant information is prioritized and becomes perseverative (i.e. extended and repetitive). The most common types of perseveration include worrying or ruminating (brooding) on negative and threatening events such as how to deal with future threats or trying to understand past events and feelings. In addition to worry and ruminations, the CAS is also comprised of attentional strategies of “threat-monitoring” such as checking for symptoms or thoughts or scanning the environment for specific signs of danger (e.g. contamination or personal rejection). Added to these elements are other forms of problematic behavior such as avoidance, inactivity, thought suppression, or substance use. These strategies intensify and extend negative processing. They also reduce direct experiences of discontinuation of processing by the mind itself.

An illustration of the CAS and its effects can be seen in a depressed patient who when questioned about feelings of lethargy reported: “I don’t have the strength to cope” and described how subsequently he responded to this cognition by analyzing why he lacked energy, compared himself with other people, repeatedly questioned why he felt depressed, closely monitored his feelings of fatigue, engaged in self-criticism in an attempt to increase motivation, and reduced activity levels in order to conserve strength. This constellation of responses prolonged negative self-focused processing and undermined his subjective ability to deal with situations.

In the S-REF model, the CAS is caused by the individual’s metacognitive knowledge (Wells and Matthews, 1994, 1996), and such knowledge is formulated as a major target in metacognitive therapy (Wells, 1995, 2000). A distinction is made between declarative and procedural metacognitive knowledge. The declarative can be expressed verbally as beliefs about thinking (e.g. “worrying is harmful”), whilst procedural knowledge exists as implicit instructional information (i.e. commands or “plans”) that inform the cognitive system how to operate (e.g. the instructions behind generating worry or rumination).

The declarative metacognitive beliefs in psychopathology can be further divided into those that are positive or negative. The positives concern the usefulness of CAS strategies such as worry, rumination, and attending to threat (e.g. “Worrying means I’m always prepared”), while the negatives concern the uncontrollability and harmfulness of cognition (e.g. “I have lost control of my thinking” and “Some thoughts can harm me”). The latter are considered of greater causal significance in disorder because beliefs concerning the uncontrollability and danger of cognition interfere with effective control and lead to omnipresent threat from an internal process; cognition itself (Wells, 1995).

It is evident in the S-REF analysis that the cognitive and neural architecture accommodates strategic processes such as worry, rumination, and threat monitoring that are conceptualized as serving personal self-regulatory goals and are linked to metacognition. However, many of the constructs in our model were new and therefore a research program was needed to develop tools for measuring metacognitive beliefs (Cartwright-Hatton and Wells, 1997), thought control strategies (Wells and Davies, 1994), and types of worry (Wells, 1994, 2005a) to facilitate model testing.

A significant proportion of work in this domain was enabled by developing the metacognitions questionnaire (MCQ; Cartwright-Hatton and Wells, 1997, Wells and Cartwright-Hatton, 2004), a measure of beliefs about thinking. The MCQ measures five domains of metacognitive knowledge each on a separate subscale: negative beliefs about thoughts concerning uncontrollability and danger (e.g. “When I start worrying I cannot stop”); positive beliefs about worrying (e.g. “Worrying helps me to avoid problems in the future”); cognitive confidence (e.g. “I have a poor memory”); need for mental control (e.g. It is bad to think certain thoughts”); and cognitive self-consciousness (e.g. “I constantly examine my thoughts”). These domains represent the declarative knowledge or information that individuals hold about thinking and are considered linked to the procedural knowledge or the commands of the S-REF that influence processing.

## Scientific Status of the Self-Regulatory Executive Function Model

The S-REF model emphasized common processes in psychological disorder, predicting universal, or transdiagnostic abnormalities in attention (e.g. threat monitoring), metacognition and perseveration. Consistent with this prediction, attentional bias has been demonstrated across different traits and disorders (Bar-Haim et al., 2007; Cisler and Koster, 2010; Staugaard, 2010; Techmann et al., 2010; Epp et al., 2012), and universal dysfunction in metacognitive beliefs has been shown across pathologies (e.g. Sun et al., 2017). In the next section, data on metacognitions and the CAS will be considered. Several extensive reviews of biased attention can be found in the literature elsewhere (e.g. Bar-Haim et al., 2007; Cisler and Koster, 2010; Epp et al., 2012).

### Metacognitive Beliefs

It is now reliably established that metacognitions are elevated across psychological disorders and are associated meaningfully with perseverative styles of negative thinking (e.g. worry, rumination) and emotional vulnerability as our model predicted (Cartwright-Hatton and Wells, 1997; Wells and Cartwright-Hatton, 2004; Spada et al., 2008; Nordahl et al., 2019). In a meta-analysis of 45 studies including 3,772 patients and 3,376 healthy individuals, Sun et al. (2017) showed elevated dysfunctional metacognitions across patients, with large and robust effects for beliefs concerning the uncontrollability and danger of worry and beliefs about the need to control thoughts. Of particular note, researchers have demonstrated that the metacognitions of the S-REF model appear to be stronger and more reliable predictors of psychological vulnerability and symptoms of disorder than the content of cognition (Gwilliam et al., 2004; Myers and Wells, 2005; Spada et al., 2007; Myers et al., 2009; Bennett and Wells, 2010; Bailey and Wells, 2016; Nordahl and Wells, 2017). Furthermore, change in metacognitions during treatment appears to predict positive outcome better than change in cognition (Solem et al., 2009; Nordahl et al., 2017), while pre-treatment metacognition may also impact on outcomes (e.g. Spada et al., 2009). Development of more specific metacognitive belief measures for depressive rumination, alcohol use, and health anxiety add further evidence of positive relationships between metacognitive knowledge, problematic affect, and behaviors (Papageorgiou and Wells, 2003, 2009; Spada and Wells, 2008; Bailey and Wells, 2015a). In addition, prospective studies support the role of elevated metacognition as a precedent to elevated emotion disorder symptoms (Myers et al., 2009; Yilmaz et al., 2011; Capobianco et al., 2019) and as a moderator of the effects of cognition on anxiety (Bailey and Wells, 2015b).

Experimental studies have sought to manipulate metacognitive beliefs directly to test their causal impact on symptoms. Rassin et al. (1999) tested the effect on obsessional thoughts in a non-clinical sample. Participants were led to believe that an EEG apparatus to which they were connected would detect the occurrence of the thought: “apple” and on doing so would deliver an electric shock to another participant they had just met. The participants were informed that they could interrupt the electric shock by pressing a button within 2 s after the word “apple” had surfaced in their consciousness. In a comparison condition, participants were told that the EEG could detect the thought “apple,” but no information about shocks was given. Thus, the experimental condition can be interpreted as inducing metacognitive beliefs about the power of the thought “apple” to cause an electric shock unless the participant acts to prevent it. The experimental condition resulted in more intrusive thoughts, greater discomfort, more internally directed anger, and greater effort to avoid thinking.

In an extension and modification of this paradigm, Myers and Wells (2013) selected non-patients who scored high and low on a measure of obsessional symptoms and randomly allocated them to a metacognitive belief induction or control condition. All participants were connected to a fake EEG apparatus and asked to watch a video about drinking water. Following the video, participants in the experimental group were led to believe that having thoughts about drinking would be detected by the EEG apparatus and if so a burst of white noise sufficient to startle them might be generated through headphones. The control group were informed that the EEG apparatus could detect thoughts about drinking, and they may receive a random burst of white noise sufficient to startle them. Therefore, only the experimental group were led to believe the aversive loud noise could be caused by their thoughts. Consistent with study hypotheses, participants high in obsessions in the experimental group reported significantly more intrusions about drinking, more time thinking about them and greater discomfort than high obsession participants in the control group.

Capobianco et al. (2018b) used the fake EEG paradigm to induce negative metacognitive beliefs about the importance of thoughts and explore their effects on stress responses. Participants were led to believe that an EEG device could detect negative thoughts and in the experimental condition this might lead to a burst of white noise. In the control condition, the noise was introduced as possibly occurring at random (there was no actual noise exposure in any condition). All subjects underwent the Trier Social Stress Test to induce stress symptoms that were measured across the study and during a 10-min recovery period. On physiological measures (skin conductance), no differences were observed between groups. But on self-report outcomes, participants in the experimental condition reported greater negative affect and lower positive affect in response to the stressor and maintained lower positive affect at recovery than control participants.

### The Cognitive Attentional Syndrome

Turning to data on the CAS, a substantial body of research supports negative effects of worry (see Davey and Wells, 2006) and rumination (see Papageorgiou and Wells, 2004) on stress responses, emotion recovery, and psychological vulnerability. Matthews et al. (1999) showed that test-anxiety measured at a trait level was positively related to maladaptive metacognition and worry (which together loaded on a general factor) and to style of coping. Furthermore, the effects of worrying appear to be influenced by metacognition in some contexts. In a study of performance under evaluative stress, the effects of high worry states on performance and psychophysiological outcomes were moderated by metacognition (i.e. meta-worry), perhaps reflecting the impact of metacognition on compensatory effort or resource allocation (Matthews et al., 2019). The impact of the CAS on symptoms of psychopathology has additional metacognitive moderators; high perceived attention control appears to reduce the strength of association between the CAS and disorder symptoms (Fergus et al., 2012).

Studies of individual differences in the control of distressing thoughts provide reliable support for the predicted negative effects of using CAS-related strategies and the ubiquity of strategies such as worry across different disorders and symptoms. A large number of studies have used the thought control questionnaire (TCQ: Wells and Davies, 1994). The TCQ separately assesses the use of worry and self-punishment, and other occasionally more adaptive strategies of distraction, social control, and reappraisal. As predicted, worry, and self-punishment are positively associated with psychological disorder symptoms (Amir et al., 1997; Warda and Bryant, 1998; Morrison et al., 2000; Roussis and Wells, 2006). The results of longitudinal analyses of traumatic stress symptoms suggest that they may have a causal role (Holeva et al., 2001; Roussis and Wells, 2008). While these data show that CAS is reliably correlated with symptoms of psychological disorder, the CAS is also distinguishable from other constructs such as psychological flexibility that are emphasized in other approaches such as relational frame theory (Fergus et al., 2013). Symptom correlates of the CAS observed in stress and emotional disorder generalize to psychosis confirming the universality of these relationships. In their systematic review, Sellers et al. (2017) identified 51 eligible studies among which findings confirmed specific positive relationships between central elements of the CAS and experiences of psychosis and psychological distress.

Experimental manipulations of CAS processes demonstrate effects on emotional outcomes and recovery from stress that are consistent with the S-REF. The induction of worry or rumination under laboratory settings maintains cognitive and emotional symptoms following stress exposure. In early work, pre-dating the S-REF model, Borkovec et al. (1983) showed that a brief period of induced worry led to greater intrusive thoughts during a subsequent non-worry task. Subsequently, Wells and Papagerogiou (1995) and Butler et al. (1995) studied the effects of induced brief worry and other forms of mentation after exposure to a stressful film and showed that worry increased the frequency of intrusive images most over a subsequent 3-day period. Reviews by Nolen-Hoeksema (1991, 2000) and Lyubomirsky and Tkach (2004) describe experimental and correlational studies demonstrating that ruminative thinking about the implications of depressive symptoms maintains those symptoms, impairs problem solving, and is associated with worse emotional outcomes after stressful life events. Capobianco et al. (2018a) tested whether specific CAS responses delayed recovery from stress. Participants were randomly assigned to CAS conditions or a distraction control condition and exposed to the Trier social stress test. The rate of recovery from self-report negative affect and physiological stress (Galvanic Skin Conductance) was monitored. Compared to a distraction condition, rumination appeared to impact on skin conductance indicating a prolonged recovery on this index, while worry subjects reported more immediate delayed recovery marked by an initial elevation in self-reported negative affect scores.

## Revisiting the Control of Cognition

Schneider and Shiffrin (1977) contrast *automatic processing* that is fast and reflexively triggered by inputs and runs with little or no conscious involvement with *controlled or “strategic” processing*, which requires varying quantities of attention resources, is partially accessible to consciousness and malleable. The cognitive system is configured such that stimuli continually trigger off circuits of automatic processing, but controlled processing is called when the system indicates a failure of performance or a situation involving novelty or personal importance. It is conceivable that abnormality in automatic or controlled processing could contribute to different degrees to the CAS such as selective focusing on threat or the persistence of worrying. For example, exposure to repeated traumas might sensitize processing assemblies for the initial detection of threat giving it an automatic nature. However, it seems this in itself would not explain the failure to disengage negative processing which is identified in the S-REF model as central to disorder. In the S-REF model sustained processing such as worry, rumination and threat monitoring is attributed to executive or strategic factors with metacognitions playing a key role.

Although both controlled and automatic processing are likely to operate in disorder (Matthews and Wells, 2000), evidence supporting the S-REF emphasis on strategic factors has grown. For example, Phaf and Kan’s (2007) review concluded: “the emotional Stroop effect seems to rely more on a slow disengagement process than on a fast, automatic bias” (p. 184). This conclusion fits neatly with a central hypothesis of the S-REF that psychological disorder is linked with strategic factors that are the cause of perseverative or extended negative processing. It also fits with the impact of effective treatment strategies derived from the S-REF, such as the attention training technique(Wells, 1990), which demonstrably enhance self-reported attention flexibility (Nassif and Wells, 2014), objectively measured attention disengagement (Callinan et al., 2015), and neurophysiological markers of executive control (Knowles and Wells, 2018; Rosenbaum et al., 2018).

The S-REF model elucidates an advanced “architecture” of control that involves two sets of distinctions; one between automatic and controlled processing and the other between cognitive and metacognitive systems. The distinction between cognitive and metacognitive systems is supported not only by self-report as reviewed above but also by neuro-imaging data.

In particular, a meta-analysis of 193 functional neuroimaging studies of executive functioning tasks (i.e. flexibility, inhibition, working memory, initiation, planning, vigilance) in 2,832 healthy individuals demonstrated that these tasks share a super-ordinate network involving the pre-frontal, dorsal anterior cingulate, and parietal cortices (Niendam et al., 2012). Additionally, imaging of neural activity during cognitive tasks such as decision making suggests a neural system located in the pre-frontal cortex mainly involved in metacognition and independent of a cognitive system (Qiu et al., 2018).

It is evident from these parallel developments in metacognitive and neuropsychological research that a more detailed modeling of the metacognitive and cognitive architectures supporting self-regulatory processing is needed to advance the field. Such a model must explain the dynamic relationship between metacognition and cognition and the nature of the structures, circuits, and information involved in the perseveration or disengagement of negative processing.

In the remaining sections of this paper, I outline a model of a metacognitive control system of the S-REF specifying the nature and influences of metacognitive processes that contribute to the CAS and maladaptation. I then explore the implications of the model for metacognitive therapy and for future theory and research in the area.

## The Metacognitive Control System

The Metacognitive Control System Model (MCS) introduces novel concepts^*^ alongside those that already feature in the S-REF. In Table 1 they are defined, and their functional characteristics are summarized to aid understanding.

**Table 1:** Definitions and functional characteristics of constructs in the MCS model.

Construct	Definition	Function
Cybernetic code^*^	Internal code generated by the MCS representing the status of cognition in relation to a reference	Can be used to regulate networks, support repetition of processing and bias the way cognition is experienced
Cybernetic looping^*^	Repetition of a processing operation	Maintains processing in pursuit of system goals and discrepancy resolution
Memory registers^*^	Temporary means of storing cybernetic code	A temporary buffer protecting against cybernetic code loss since the comparator is constantly transitioning to the next sequence of processing
Meta-representation^*^	Pattern of activation (e.g. sensory) in the neural net in response to cybernetic code	Provides a context for cognition that can be processed according to various goals (e.g. to be meta-aware, have an objective stance, or sense of self)
D-knowledge	Declarative knowledge about cognition usually represented as metacognitive beliefs (e.g., “Bad thoughts will make me bad”)	Provides a library of data about thinking stored in long-term memory for use in self-regulation
P-knowledge	Procedural knowledge or commands that instruct processing operations	Provides general purpose orders or “programs” to control the MCS, CS and modulate the networks
Comparator	A mechanism of the MCS that compares the current status of CS processing against a reference (e.g. goal)	Enables cognitive processing to remain on-track and errors/discrepancies to be detected
Mental Model	Active representation of current processing that contains the desired goal	Provides a benchmark for the comparator
Monitoring	Flow of information from the CS to the MCS	Updates the MCS concerning the real-time status of on-line processing
Control	Flow of information from the MCS to the CS	Biases the activity of on-line processing

**Figure 1:**
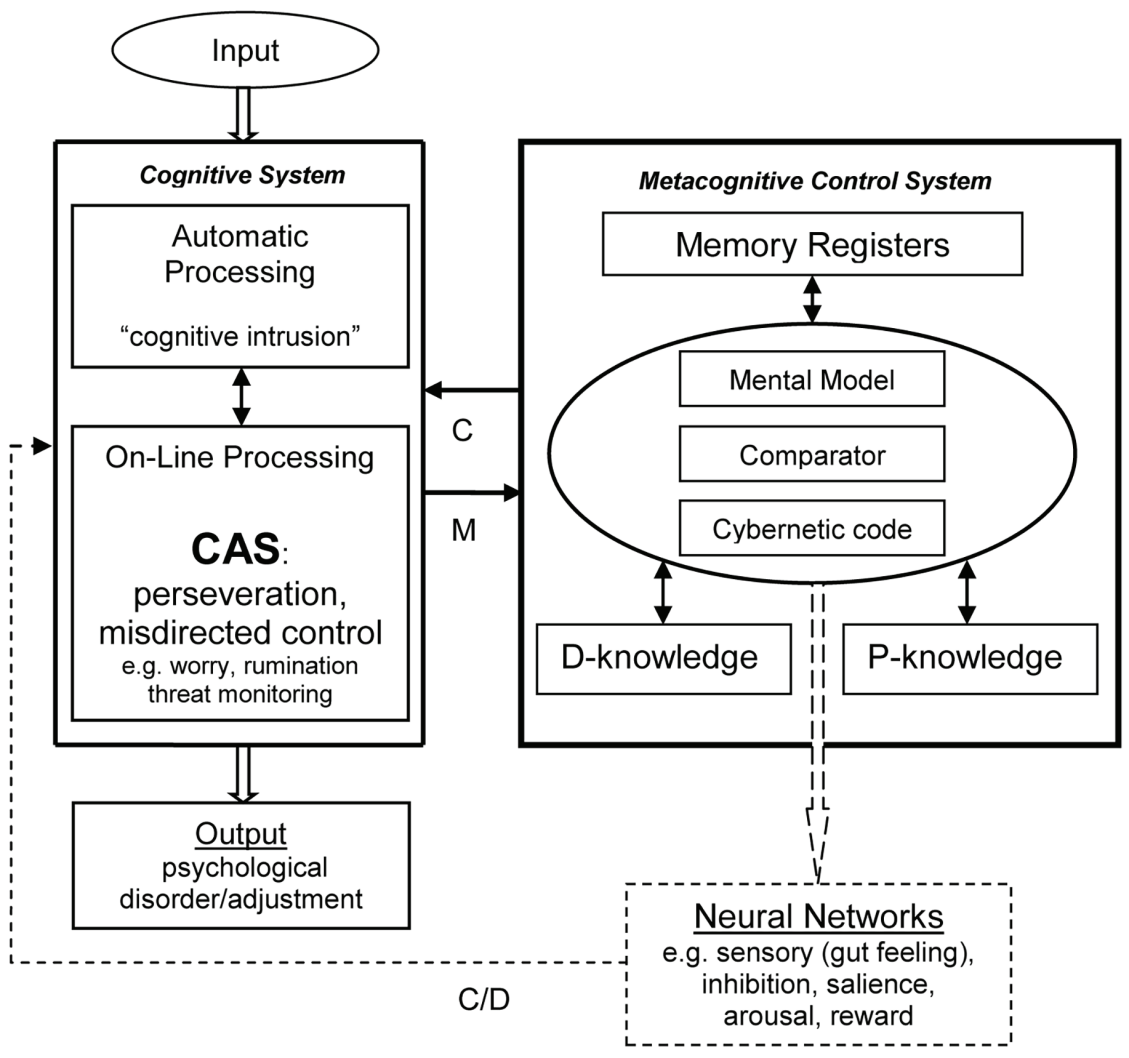
A model of the metacognitive control system and relationships with cognition. Schematic shows main components not a definitive architecture. D-Knowledge, declarative knowledge (e.g. beliefs: “Worrying is dangerous”); P-Knowledge, procedural knowledge (i.e. processing commands); C, control; M, monitoring; D, data.

A simplified schematic of the metacognitive control system (MCS) and its relationship with the cognitive system (CS) is depicted in Figure 1. Three overall sets of components are differentiated in the figure: (1) cognitive system (where automatic and on-line strategic processing are further distinguished), (2) metacognitive system, and (3) neural networks. It should be noted that this tri-partite separation simplifies the architecture and overlap and sharing of some structures and processes is expected. In particular, both cognitive and metacognitive processing are likely to consist of automatic and strategic processes but for simplicity this is not shown. The model is intended to represent features of standard architecture and processes for cognitive control, but as depicted the cognitive system (CS) is populated with the type of on-line processing (i.e. the CAS) that gives rise to psychological disorder.

The MCS is comprised of a comparator mechanism, metacognitive information in the form of declarative knowledge (D), procedural knowledge (P), and cybernetic code. There are also temporary memory registers. Different types of on-line processing are directed by the MCS, not just the style of extended negative processing that constitutes the CAS.

The function of the MCS is to monitor (M) and control (C) the activities of the cognitive system in pursuit of processing goals. It achieves this through direct and indirect effects involving the flow of information *via* the circuits depicted.

The cognitive system, shown in the left-hand side of Figure 1, is comprised of low-level automatic processing and on-line (strategic) processing that includes the limited capacity “thinking space.” The output illustrated is labeled “psychological disorder” and is considered the consequence of the cognitive attentional syndrome (CAS) dominating on-line processing as depicted. Under different on-line processing configurations, where, for example, inhibition of worry under control of the MCS is specified, internal psychological events will be transitory and therefore not constitute “disorder.”

Some features of metacognitive control are attentionally demanding and require conscious involvement and therefore draw on limited capacity processing which may compete with CS on-line processing. The operations of the MCS depend on temporary and longer-term memory stores, with some specialized memory structures (i.e. memory registers) among other dimensions (e.g. those involved in comparator function) likely to be specific to the MCS.

Centrally, the MCS continuously monitors and tests through the comparator mechanism the current state of processing in the CS against an internal model. The model represents a reference standard for the present and future/expected state of cognition. After a discrepancy or mismatch (error) is detected, instructions are issued to control mechanisms to bring CS processing in-line with goals. To accomplish this control function, it is hypothesized that the MCS has a capability to translate the current status (e.g. a discrepancy) into information; a *cybernetic code* that can be used to influence the behavior of cognitive and neural systems, biasing activity toward, for example, discrepancy reduction. It is therefore hypothesized that an important function of the MCS is generating, storing and using cybernetic information in the control of processing.

Code can influence processing across different neural networks that are recruited to bias the CS. For example, the code may be used to send commands to interoceptive networks leading to a “felt-sense” or “gut-feeling” that is recruited to bias or maintain a particular processing routine. As a means of illustration, consider an experience familiar to most people; the “tip-of the tongue” effect. When an item cannot currently be retrieved from memory (a discrepancy), this is accompanied by a strong somatic feeling and repetitive and sustained retrieval attempts that are often strategic but can also continue autonomously long after the individual has given up trying to remember. Thus, in this example, production of interoceptive responses and changes in arousal linked to receiving a signal of discrepancy (code), bias retrieval (perhaps a type of state-dependency effect), maintain implementation of retrieval instructions and increase motivation for sustained strategic memory search.

Because the comparator is consistently transitioning to the next set of processes, the system must protect against the loss of earlier code when the goal of processing remains unmet. A solution is for code to be stored temporarily in *memory registers*. It is then available to the system for repeating processing sequences – *cybernetic looping* – in pursuit of goals. Cybernetic looping, or repetition of a set of processes, like in the example of sustained memory search in the “tip-of-the tongue” experience is usually adaptive. Looping increases the probability of goal attainment (e.g. memory retrieval).

An important question relating to self-regulation concerns the determinant of number of repetitions of a cognitive process (i.e. adaptive perseveration) in an attempt to reach processing goals, especially when goals are unattainable. Several possible solutions to this issue need to be explored. It seems most probable that there are in-built system limits to iterations of processing, which may continue until neuronal or biological states (e.g. level of arousal) change. Plausibly, the memory registers holding cybernetic code may be temporary with decay being the norm. These proposed characteristics may be an important feature of psychological recovery or adaptation that naturally ensues over time. Nevertheless, this process could be adversely affected by dysfunctional metacognitive knowledge (e.g. “I must worry about all negative possibilities” or “I have lost control over thinking”). Under these influences choice of self-regulation strategy is dominated by the CAS (e.g. worry), which perpetuates processing and contributes to discrepancies (e.g. a sustained sense of threat).

This and other important implications emerge from the cybernetic code hypothesis. Under the direction of commands presented in procedural knowledge, cybernetic code could be used to control processing at different destinations in the neural network. For example, when specific commands activate or bias interoceptive processors it becomes *via*ble to “somatize” or feel the status of cognition. Feasibly, through this function the “sensing” of discrepancies and perhaps other mental processes can be implemented by the procedures of the MCS. In consequence, this allows for more complex internal representation and communication of the events occurring within the CS. A “sensing” of cognition may be a building block of the *embodiment of thinking* and a process likely to be important in the construction of self-awareness, to which I will return later.

As I have already proposed a range of memory structures are required to make *internal cybernetic communication* possible and are depicted as part of the MCS in Figure 1. There must be temporary storage (i.e. memory registers), long-term stores of metacognitive declarative (D-knowledge), and procedural (P-knowledge). While the memory registers act as a temporary buffer to protect against cybernetic code loss, the long-term memory stores provide metacognitive information and the instructions or commands for the model, the comparator process, and control of other neural systems.

### Embodiment and Self-Awareness

The theoretical structures and inter-relationships described above provide an architecture, set of functions, and feedback systems that could have several useful properties. They enable real-time information about cognitive activity to pass *via* monitoring into the MCS. In turn, under the commands of procedural knowledge, cybernetic code about cognition can be generated and influence processing in specific networks. Depending on the networks involved a combination of interoceptive (arousal), visual, or auditory processing activity linked to the code can arise. This raises the possibility that metacognitive commands (procedural knowledge) could specify that processing activity in particular networks is used as data (D in Figure 1) to create a context or *meta-representation* for the events in on-line processing. A system of such configuration could be directed by its procedural knowledge to compute in on-line processing a particular *meta-representation* consisting of a subjective stance in relation to cognition as objectifiable, separate from external events and within (i.e. tangible, felt, or embodied). Such a mechanism might provide a basis for states of objective meta-awareness (i.e. a “sense of cognition” e.g. a *feeling* that an item of knowledge is stored in memory). Furthermore, if procedural knowledge or system commands specify that objective meta-awareness (i.e. the “sense-of-cognition”) is processed symbolically as “I” or “me” within on-line processing, objective *meta*-awareness is transformed into *self*-awareness. Thus, self-awareness as conceived may require as a building block a basic metacognitive system configuration within which the commands generate a sensorial response to cybernetic information which is subject to “on-line” (i.e. conscious) symbolic processing.

A propensity to experience meta-awareness, to objectify thoughts and memory and label the observer as “self” creates enablers and barriers to cognitive control. Self as a construction or context for cognition provides for greater flexibility and development of control because it permits cognition to become the object of focal attention and the subject of an individual’s motivations and goals. For example, a person’s explicit goals can be to improve problem solving, concentration or memory ability, or to become more optimistic. What is more, it means that the private content of cognition can be shared and modified through language or other forms of expression. Ironically, it also means that private cognition can be hijacked and underlying metacognitions corrupted by, for example religious and social systems that sanctify or punish the possession of certain thoughts and beliefs.

## Treatment Implications

The ideas developed in this paper are the basis of metacognitive therapy (MCT), which focuses on reducing the CAS and modifying metacognition so that recovery can occur. Full MCT treatment was first developed for generalized anxiety disorder (Wells, 1995, 1997) and subsequently other disorders (Wells, 2000, 2009). In meta-analyses, MCT demonstrates large treatment effects and appears potentially more effective or more efficient than cognitive behavioral approaches (Normann et al., 2014; Normann and Morina, 2018). In a direct test of transdiagnostic MCT against disorder-specific CBT across anxiety disorders, outcomes favoring MCT were reported (Johnson et al., 2017) and potential mechanisms of change could be distinguished (Johnson and Hoffart, 2018). Several trials have evaluated the effects of MCT against CBT for generalized anxiety. In each case MCT was superior (Van der Heiden et al., 2010; Wells et al., 2010; Nordahl et al., 2018). More naturalistic studies of less highly selected patients also support positive treatment effects of the full MCT package (e.g. Hagen et al., 2017; Papageorgiou et al., 2018; Callesen et al., 2019) and of individual treatment techniques (e.g. Knowles et al., 2016). The majority of treatment outcome studies have been conducted in anxiety and depression, but preliminary feasibility data suggest that the treatment can be implemented in psychosis (Morrison et al., 2014; Carter and Wells, 2018), transdiagnostic group settings (Capobianco et al., 2018c), comorbidity (Hjemdal et al., 2017), treatment resistant cases (Wells et al., 2012; Winter et al., 2019), alcohol abuse (Caselli et al., 2018), and traumatized borderline personality (Nordhal and Wells, 2019).

### Advanced Treatment Considerations

What is the impact of the MCS model for clinicians and researchers aiming to develop a better understanding of the mechanisms and processes of MCT and its effective practise?

A consequence of separating the cognitive system from the MCS in conceptualizing information processing is the following: worry, rumination, appraisals, and the execution of behaviors are all processes occurring within the cognitive system (CS). However, control, executive processes, knowledge supporting control and information on the current status of cognition are properties of the MCS. In psychological disorder it is chiefly the MCS that is the cause of bias observed in the cognitive system (CS). Maladaptation in the MCS is the major internal source of extended negative processing (the CAS) occurring in the CS. An implication of the distinction is that treatment should focus on formulating and modifying the content, strategies, and regulatory influence of the MCS as the most important source of disorder. Thus, treatment does not as a matter of emphasis focus on changing the properties of the CS such as the content of thoughts, general beliefs, memories or images or aim to change reflexive (automatic) networks of the CS through prolonged exposure techniques.

The conceptualization of procedural metacognition located in the MCS and its separation from cognition (the CS) presents an important implication concerning how treatment is conducted. It means that MCS knowledge; not only declarative but also the procedural commands that direct the comparator and bias the activities of CS must be extracted from the MCS and processed (e.g. modified) in the CS on-line before being returned to the MCS or sent to another location in the network. Crucially, this means that the appropriate parcel of procedural knowledge must be extracted; that which is the source of the CAS. Since the CAS can take a variety of forms the therapist must accurately identify it on a case by case basis. Furthermore, excessive CAS activity in the CS must be moderated early in therapy, so that the limited capacity “thinking space” can be liberated and used for MCS modification.

Metacognitive therapy contains techniques designed for the above purpose that explicitly induce and “hold” the patient in a “metacognitive mode” of processing during sessions with the aim to modify both declarative and procedural meta-knowledge while governing CS processing load. These techniques include among others: meta-level discourse, the attention training technique, the free-association and tiger tasks, rumination postponement, metacognitive focused exposure, metacognitive experiments, and worry-modulation procedures. The therapist must use direct *metacognitive experiences* and a *discourse* that transforms processing styles in the CS before reassigning the knowledge supporting them to the MCS. In this manner, the techniques used increase the range, choices, and flexibility with which the individual controls and can relate to their CS. These techniques are described in detail elsewhere (Wells, 2005b, 2009).

The model highlights clear differences between metacognitive therapy and other treatment approaches in the intended target of change. In MCT, the therapist retrieves and modifies the validity of declarative metacognitions and also retrieves and re-writes the commands (procedures) for regulating processing with the purpose of modifying those involved in the CAS. In contrast, other treatments either do not aim to work on metacognitions or they do so without maintaining a clear structural and functional distinction between systems. But such a distinction could be facilitative in the design of more advanced theory-grounded treatment techniques. For example, if we consider the treatment of low self-esteem, a cognitive therapist will aim to identify and challenge negative beliefs about the self by asking questions such as: “What is the evidence you are a failure, is there another way to view the situation?” but the metacognitive therapist would ask: “What’s the point in analyzing your failures?” and follows with techniques that allow the individual to directly step-back and abandon the perseverative thought processes that extend the idea. Of particular importance, in MCT, the client discovers that processing remains malleable and subject to control in spite of the dominant cognition (belief) “I’m a failure,” thus creating an alternative model of processing rather than an alternative model of the social self (the latter considered a secondary topographic event).

Good metacognitive therapy, the model suggests, is that which modifies the procedural knowledge base. It should enable the individual to: (1) directly alter the relationship or “stance” they have with products of cognition; (2) directly manipulate the control of cognition (e.g. delay worry and inhibit perseverative thinking); and (3) separate metacognition (i.e. mechanisms of control) from the strong influence of internal (e.g. thoughts and feelings) and external events (as per Attention Training Technique protocol). The systematic regulation of attention using a framework of discovery that shows attention remains flexible irrespective of mental events supports the development of general-purpose strong metacognitive control procedures of this kind.

An implication of the MCS as described is that it can (under commands of procedural knowledge) initiate and hold in the moment different *meta-representations* of internal cognition. A meta-representation is influenced by the effect of the current cybernetic code on other processors that provide input to on-line processing. This creates flexibility and the possibility of choosing how to relate spatially and sensorially (or emotionally) to inner thoughts, memories and mental events. In *object mode,* thoughts are experienced as direct perceptions and treated as facts (the individual is in the thought), but in *metacognitive mode,* they are experienced as events or stimuli in the mind and the individual steps outside of them (Wells and Matthews, 1994). The model directs us toward developing techniques that change the *meta-representational state*. For example practise of “flipping” between modes or of co-joint experiencing of incongruent thoughts (e.g. negative thought plus positive memory) or of experiencing a negative thought and coupling it with a positive feeling. In each case the meta-representation might be changed by shifting “stance” or coupling cybernetic code with new and incongruous bodily and affective states.

Since a goal of MCT is to reduce over-reliance on thinking, it is usually better to shift into a metacognitive mode and disengage further conceptual processing rather than analyze and interrogate negative thoughts as a means of change. However, the model suggests that an exception must occur when a negative metacognitive appraisal or meta-belief is present (e.g. “Worrying will cause cancer”). Since this is primarily a property of the MCS (it reflects maladaptive metacognitive knowledge), it should be evaluated and replaced with more adaptive information because it will continue to impact on cognitive control and the stance in relation to cognition. To summarize, in metacognitive therapy challenging of the validity of metacognitions is supported, but challenging the validity of cognitions is not.

### Metacognitive Focused Exposure

Simply engaging the CS in activities of cognitive-behavior therapy such as evaluating the validity of thoughts or repeated exposure to fear stimuli present imprecise and coincidental ways of modifying the control system. Exposure is considered to facilitate habituation or “emotional processing,” which is defined as: “a process whereby emotional disturbances are absorbed and decline to the extent that other experiences and behavior can proceed without disruption” (Rachman, 1980, p. 51). This has typically been viewed as a mechanism whereby information about declining arousal is automatically incorporated in fear networks (e.g. Foa and Kozak, 1986) such that pre-existing links between stimulus-response nodes and negative meanings attached to anxiety are weakened. This conception of emotional processing relates most closely to automatic processing and neglects the involvement of upper-level cognitive structures, including the metacognitive control system. For example, it is possible to think about an emotional event in an unemotional way. Furthermore, the network approach does not address questions concerning the factors that determine the cessation of emotional processing or how the goals of emotional processing are represented and monitored?

The MCS model invites the clinician to concentrate treatment on top-down influences on extended processing such as the use of worry, over-analysis of memory or threat-monitoring that lead to repeated or sustained activation of fear networks. The MCS model also implies that emotion networks may respond to cybernetic code and the impact of code on the network may be moderated by metacognitive knowledge. For instance, the ability to think about an emotional event in an un-emotive way is resolved, because the MCS can change the nature of the relationship (meta-representation) with thoughts. In addition, theoretical questions about the cessation and representation of the goals of emotional processing are dealt with by hypothesizing that the MCS can monitor and control emotional networks partly through its comparator and cybernetic code functions. Emotional processing stops when the goal of processing is met or when the cybernetic code decays. The ability to achieve such exit signals is potentially reduced by the CAS and dysfunctional metacognitions, leading to psychological maladaption.

There are implications of the model for developing more efficient and effective exposure therapy techniques. This can be achieved by inhibiting the CAS during exposure and by configuring exposure to explicitly modify maladaptive metacognitive knowledge; both declarative and procedural. Such an approach of *metacognitively focused exposure* has been previously introduced (Wells, 2000).

In a simple form, the combination of exposure with attention instructions designed to reduce threat monitoring and increase access to non-threat related information will be helpful. But more unexpected applications are indicated. For instance, the MCS model presents an idea that runs counter to the traditional approach to exposure treatments that emphasize the need to eliminate avoidance. If we take as an example the treatment of obsessive-compulsive disorder, exposure and prevention of covert and overt rituals (forms of avoidance) such as repeated washing is an effective and recommended treatment. In contrast to this approach, in MCT, the patient can be permitted to use rituals in response to thoughts provided they hold the thought in mind, because the goal is to change the meta-representation of the thought in the MCS and not the associative links at a fear network level through habituation. The aim in MCT is to change the nature of the person’s relationship with negative cognitions so that *thoughts are experienced* as unimportant and transient events in the mind.

A small number of pilot studies have experimented with forms of metacognitive focused exposure. Fisher and Wells (2005) examined the effects of brief exposure when it was presented as an experiment to explicitly test metacognitive beliefs in OCD. In this study, patients with OCD were asked to listen for 5 min to their obsessional thoughts recorded on a loop-tape under two contrasting conditions. In one condition, a habituation instruction was used with the goal of staying with the feelings of anxiety and stopping any rituals. In the metacognitive condition, the instruction was also to stop any rituals but with the goal of discovering that the thoughts were unimportant. While both rationales were seen as equally credible by participants, the metacognitive condition was associated with significantly greater reductions in anxiety, metacognitive beliefs and urge to neutralize. In another study, Wells and Papageorgiou (1998) exposed social phobia patients to feared social situations under a habituation rationale or external attention focusing rational that counteracted threat monitoring. The latter condition produced superior effects after a single brief exposure.

### Resistance to Change

The present model offers a means of understanding and dealing with resistance to change in psychotherapy. It implies that metacognition can act against a person “changing their mind.” The model draws the clinician to the paradoxes in cognitive control such as holding both positive and negative metacognitive beliefs concerning sustained processing. In generalized anxiety disorder (GAD), the client believes that worrying will help anticipate and avoid threat but in conjunction with this there is the belief that worrying is uncontrollable and harmful (Wells and Carter, 2001). In health anxiety, there is a belief that negative misinterpretation of symptoms will facilitate illness detection and also that thoughts can cause illness (Bailey and Wells, 2015a). In depression that analyzing why one feels depressed will lead to feeling better but might also cause self-harm (Papageorgiou and Wells, 2001, 2003). Each of these examples presents potential ambivalence, uncertainty, or vacillation in abandoning the CAS. A belief in the uncontrollability or pure “biological basis” of negative cognition contributes to a sense of hopelessness, reduced effort invested in control or a reliance on extraneous forms of control. This acts against the client using their own internal control, which might otherwise enhance MCS capacity to create change.

We have seen how a proposed normal in-built mechanism; cybernetic looping, contributes to perseveration of processing. This could explain persistent but relatively normal affective and motivational states such as longing, desire, grief, craving, anger, regret, shame, and remorse among others. In these instances and in stress and adjustment reactions, we would expect spontaneous recovery over time. However, when an individual uses the CAS as a coping strategy it maintains the sense of threat and disrupts the normal exit conditions for the cybernetic loop, leading the individual to become “gripped” by their feelings. Furthermore, worrying and ruminating consume processing resources that are required for metacognitive control such as switching between goals for processing, consequently negative processing is less flexible and persists. In each of these cases, the treatment aim should be to remove the barriers (i.e. CAS) to exit and effective internal control conditions. Usually, perseverative processes appear to have an in-built limited and system determined repetition that we might conceptualize as a normal psychological recovery period. This concept is used in treating post-traumatic stress disorder, where the explicit goal shared with clients in MCT is to remove the CAS so that in-built *reflexive adaptation processes* run their natural course (Wells, 2009; Wells and Colbear, 2012; Wells et al., 2015). An important implication is that restructuring thoughts about trauma, modifying trauma memory and reliving methods are not necessary for effective treatment. Treatment should only be introduced after recovery processes have been given an opportunity to run naturally.

Cognition is not supplied with a user manual or a schematic that allows the owner to understand how it works or how best to operate it. However, we rely on information and procedures (knowledge) of how our memory and attention works, we learn to compensate for tiredness or a noisy environment by increasing effort or concentration, we learn what a thought is, what a dream is, that we have a good memory for places, and that cognition is harmless and not prone to loss of control. We might reasonably assume that metacognitive knowledge about cognitive control has a special place and powerful influence on how we construe our own experiences and how much we allow our own mental events to impact and shape our lives. The impact can be profound. For instance, consider how some approaches to mental illness might contribute to a disabling and unhelpful knowledge of metacognitive control that solidifies a sense of helplessness and mental brokenness. This is not very useful to the individual, but the discovery of control and a belief that recovery is a matter of letting some thoughts go is likely to be more beneficial. More broadly, the MCS model encourages us to examine the messages carried by existing approaches to mental health diagnosis and treatment. Treatment delivery programs should ensure that unhelpful metacognitions are not created but those that already exist are modified.

### The Process of Recovery

Implicit in all that I have described above is a fundamental idea. The MCS is involved in the perpetuation of negative psychological experiences, and it is also involved in their cessation; it plays a role in recovery. Under typical circumstances, we might consider the cybernetic code functions as a “code for recovery” because it supports continued processing toward goal attainment and any repetition of processing is usually limited. However, when metacognitions specify the CAS and when they give rise to a sense of uncontrollability and threat from cognition itself, errors or deviations from reference internal states persist and the code is constantly refreshed. The process of recovery in psychological therapies is one in which decay of the code and exit conditions for cybernetic looping are made accessible. In MCT, this is achieved through modifying maladaptive metacognitive knowledge, by enhancing flexible control and by disengaging the coping strategies that depend on extended processing.

## Limitations and Future Research

It must be borne in mind that the model is rudimentary and a project in development. For example, in the interests of simplicity I have shown “automatic processing” as a separate cell in Figure 1. However, a dichotomy between automatic and controlled processing is simplistic, and it may be better to view processing along a continuum of automaticity across multiple systems. Some automatic processes in the CS may prime specific procedural knowledge within the MCS, so the CS has some limited influence over the MCS, which is not explored. The CS is controlled by its own “hard-wiring” and in a more flexible and extended way by the procedural knowledge and codes of the MCS. The processes of the MCS, such as activities of the comparator and the priming of procedural knowledge are unconscious and the processes reflexively “run-off” in response to stimuli.

Unanswered questions surface concerning the reliance of both metacognition and cognition on shared and domain-specific structures and processes, among them memory. In particular, depiction of the memory registers is not intended to imply that these are structurally equivalent to long-term memory or working memory. Instead, the model points to the importance of exploring and separating multiple components of memory including the hypothesized memory registers and processes that temporarily represent discrepancies in processing. The prediction that activity in such structures and related processes is moderated by cybernetic code offers a potential means to distinguish them from other memory processes using paradigms that induce code (i.e. cause discrepancies such as violations of expectancy and induction of performance errors).

There are clear limitations in the current database, including a paucity of information concerning the antecedents of dysfunctional metacognitive knowledge, such as the possible role of stressful early life experiences (e.g. Myers and Wells, 2015). Furthermore, while preliminary evidence suggests that different components of metacognitive knowledge may interact in explaining distress, this remains to be explored in detail. For instance, interaction between knowledge about attention and beliefs about uncontrollability of thoughts appears to provide additional nuanced effects (at least in children) that may prove important (e.g. Reinholdt-Dunne et al., 2019).

So far in this account I have intentionally avoided any detailed consideration of the detrimental effects of metacognition on performance of cognitive tasks. The detrimental effects of anxiety on performance are well established (e.g. Eysenck, 1992). Anxious mood appears to be a stronger determinant of impaired performance than trait-anxiety, with worry predicting poorer performance better than emotional and physiological aspects of anxiety (e.g. Morris et al., 1981). Eysenck and Calvo (1992) proposed that anxiety impairs the efficiency of the central executive which appears much like working memory as proposed by Baddeley (1986). Their theory assumed that task-irrelevant processing such as worry does not always have a negative impact on the effectiveness of performance. Finding oneself worrying may in fact enhance motivation to overcome the negative performance effects by using additional processing resources. This appears to be at odds with the idea of a CAS that causes problems. However, it remains consistent with the MCS model because the ability to compensate will depend on characteristics of the MCS. In particular, metacognitive beliefs of lack of control should negatively influence the level of compensatory resources used. For example, in a study by Matthews et al. (2019), the effects of high worry on performance and neurophysiology under social-evaluative stress was dependent on the level of meta-worry (i.e. negative appraisals of the uncontrollability and danger of worrying).

It remains to be determined how the MCS might relate to a wider range of executive functions, to concepts such as working memory (Baddeley, 1986, 1996) and inhibition and attention shifting functions hypothesized by Eysenck et al. (2007) in attention control theory. But the model points to the importance of examining the influence of metacognitions on these dimensions.

While there is strong evidence of dysfunctional metacognitive knowledge across psychopathologies, most of the evidence is at the level of self-report. Self-report can be criticized, but it is a mistake to dismiss it as it provides important clues to the consciously accessible aspects of information processing such as goals and choice of strategy. But this area of research needs to be strengthened by investigating further the effect of self-report metacognitions on attentional responses at a performance and neural level. Such efforts should seek to explore the cybernetic code hypothesis and map the neural structures, circuits and dynamic effects involved. Usefully, the MCS model suggests the development of laboratory paradigms to probe and isolate such effects by using the induction of discrepancies between actual and desired processing states, such as violating cognitive expectancies. If a trace of the cybernetic code in such paradigms can be detected in the form of activity or temporary change at a cellular or network level this might be used as proof. It may be possible to adapt this, using speed of decay of such activity produced in discrepancy induction paradigms to measure inherent psychological resilience. For example, greater resilience might be associated with faster loss of the cybernetic code from memory registers.

Finally, the model presents important questions and research directions concerning childhood development of the MCS; when and what are the influences on the development of beliefs about inner-thought? Is there a sequence of development of attention control skills and is there an optimal set pattern? We might hypothesize that it is possible to identify *proto-metacognitive* states and stages that track the transition from early attention fixation and limited control through to acquired attention flexibility and the later development of higher-order knowledge of control necessary in consolidating a MCS. Exploration of levels of complexity and degree of inter-connectedness of the CS and MCS presents major trajectories for future cognitive and neuropsychological research.

## Conclusion

The S-REF model has influenced research on cognitive control in psychological disorder, placed top-down processes and metacognition in a prominent role and informed the development of metacognitive and other therapies. But an important challenge remains: to strengthen the theoretical foundations necessary to advance the study of metacognition in self-awareness and mental health. One means is by exploring and describing in detail the components, architecture and functions of the metacognitive control system of the S-REF and how it relates to disorder; my goal in this paper. In particular, the field can benefit from consideration of the types and effects of metacognitive information generated and used by the system in pursuit of cognitive regulation. This has become more justified as evidence from neuropsychological and S-REF based research supports a neural system separate from cognition and involved in metacognition as the S-REF predicted.

Psychological disorder from the position of the S-REF model is conceptualized as a state of persistence of negative processing that is difficult to control. In most cases, negative ideas and feelings are transitory but in psychologically vulnerable individuals they become extended and “fixed” due to a transdiagnostic style of thinking: Cognitive Attentional Syndrome (CAS). The CAS is largely a consequence of the impact of biased metacognitions on cognitive regulation. Persistence of processing is influenced by different features of the MCS; repetition of processing is normally a feature of cybernetic looping when discrepancies or errors are detected. But in psychological disorder this effect is disrupted by choice of strategies linked to metacognitive knowledge that interfere with exit conditions for looping, diminish inhibitory control attempts (e.g. “I have lost control of my thoughts”) or sanction extended processing (e.g. “I must analyze all my failures until I become a success”).

An architecture replete with metacognitive information (i.e. declarative and procedural knowledge, mental models, cybernetic code and metacognitive experiences) has emergent properties that contribute to cognitive control. It is a framework for the development through meta-representational states of within-ness (embodiment), self-awareness, and a subjective ownership of cognition. Such effects normally increase flexibility, a sense of stability, and self-control of thoughts. They also facilitate the social communication of thought, but they can as described present a wider range of potential loci for bias that contributes to disorder. At the most basic of applied levels, health systems and clinicians working with service users must begin to consider the potential negative effects on metacognition of the information and treatment techniques they provide.

In the future, it may be possible to describe the proposed psychological structures and processes with greater precision. But for now the model points to the potential in isolating a discrete metacognitive control system that is separate from cognition, studying the impact of its components and content on psychopathology, self-awareness, and self-regulation. I have described how strengthening this separation can continue to provide a basis for theoretically derived treatment techniques in MCT that target specific causal mechanisms in a particular way. The MCS model opens up a substantial set of new avenues for research addressing issues that include: mapping the role of different neural systems in cognitive control; testing the effects of discrepancies or violations of expectancies (i.e. production of cybernetic code) on interactions between systems; testing the co-dependence of metacognitive and cognitive operations on limited capacity; examining the multiple memory requirements and processes of metacognition; testing the interactive effects of metacognitive knowledge and attention control on symptoms; exploring the relationship between metacognition and self-awareness; and in a broad context examining untoward effects of healthcare delivery and social systems on metacognitive functioning. It provides a framework for a more unified cognitive, social and neurobiological theory of awareness, self-regulation and mental wellbeing.

Advances in psychotherapy require a paradigm shift; stronger information processing theory that can successfully explain the control of cognition and the negative subjective changes in perceived control and sense of self that are central features of disorder. Psychological wellbeing is not a matter of what we think. It is an issue of how we regulate the cognitive processes that prioritize and extend thoughts. It is the stance taken in relation to the content of the limited capacity “thinking space.” It is above all, the nature and effect of *metacognitive information* generated, held and used by processing systems.

## Author Contributions

The author confirms being the sole contributor of this work and has approved it for publication.

### Conflict of Interest

The author declares that the research was conducted in the absence of any commercial or financial relationships that could be construed as a potential conflict of interest.

The reviewer GC declared a past co-authorship with the author AW to the handling editor.
